# Decoding LncRNA in COPD: Unveiling Prognostic and Diagnostic Power and Their Driving Role in Lung Cancer Progression

**DOI:** 10.3390/ijms25169001

**Published:** 2024-08-19

**Authors:** Osama Sweef, Reda Mahfouz, Tülin Taşcıoğlu, Ali Albowaidey, Mohamed Abdelmonem, Malek Asfar, Elsayed Zaabout, Yalitza Lopez Corcino, Venetia Thomas, Eun-Seok Choi, Saori Furuta

**Affiliations:** 1 Division of Cancer Biology, Department of Medicine, MetroHealth Medical Center, School of Medicine, Case Western Reserve University, 2500 MetroHealth Drive, Cleveland, OH 44109, USA; 2 Department of Zoology, Faculty of Science, Tanta University, Tanta 31527, Egypt; 3 Core Laboratory, University Hospital Cleveland Medical Center, Department of Pathology, School of Medicine, Case Western Reserve University, 1100 Euclid Avenue, Cleveland, OH 44106, USA; 4 Department of Clinical Pathology, Faculty of Medicine, Menofia University, Shebin-Elkom 32511, Egypt; 5 Department of Molecular Biology and Genetics, Demiroglu Bilim University, Esentepe Central Campus, Besiktas, 34394 Istanbul, Turkey; 6 The Ragon Institute of Mass General, MIT, and Harvard, Cambridge, MA 02139, USA; 7 Department of Microbiology, Immunology, and Cell Biology, School of Medicine, West Virginia University, Morgantown, WV 26506, USA; 8 Department of Pathology, Transfusion Medicine Service, Stanford Healthcare, Stanford, CA 94305, USA; 9 Department of Pathology, MetroHealth Medical Center, School of Medicine, Case Western Reserve University, 2500 MetroHealth Drive, Cleveland, OH 44109, USA; 10 Department of Therapeutics & Pharmacology, The University of Texas MD Anderson Cancer Center, UTHealth Graduate School of Biomedical Sciences (GSBS), Houston, TX 77030, USA

**Keywords:** chronic obstructive pulmonary disease (COPD), long non-coding RNAs (lncRNAs), molecular pathogenesis, inflammation, lung cancer, diagnostic biomarkers

## Abstract

Chronic obstructive pulmonary disease (COPD) and lung cancer represent formidable challenges in global health, characterized by intricate pathophysiological mechanisms and multifaceted disease progression. This comprehensive review integrates insights from diverse perspectives to elucidate the intricate roles of long non-coding RNAs (lncRNAs) in the pathogenesis of COPD and lung cancer, focusing on their diagnostic, prognostic, and therapeutic implications. In the context of COPD, dysregulated lncRNAs, such as NEAT1, TUG1, MALAT1, HOTAIR, and GAS5, emerge as pivotal regulators of genes involved in the disease pathogenesis and progression. Their identification, profiling, and correlation with the disease severity present promising avenues for prognostic and diagnostic applications, thereby shaping personalized disease interventions. These lncRNAs are also implicated in lung cancer, underscoring their multifaceted roles and therapeutic potential across both diseases. In the domain of lung cancer, lncRNAs play intricate modulatory roles in disease progression, offering avenues for innovative therapeutic approaches and prognostic indicators. LncRNA-mediated immune responses have been shown to drive lung cancer progression by modulating the tumor microenvironment, influencing immune cell infiltration, and altering cytokine production. Their dysregulation significantly contributes to tumor growth, metastasis, and chemo-resistance, thereby emphasizing their significance as therapeutic targets and prognostic markers. This review summarizes the transformative potential of lncRNA-based diagnostics and therapeutics for COPD and lung cancer, offering valuable insights into future research directions for clinical translation and therapeutic development.

## 1. Introduction

COPD represents a formidable global health challenge, characterized by a progressive and irreversible decline in airflow, caused by chronic inflammation and other types of respiratory symptoms [1]. Principally associated with prolonged exposure to noxious particles, notably tobacco smoke, COPD imposes a substantial burden on individuals and healthcare systems worldwide [2]. Recent years have witnessed heightened scrutiny of the intricate molecular mechanisms underpinning COPD pathogenesis. Among the diverse array of factors contributing to its etiology, lncRNAs have emerged as pivotal regulators of gene expression and cellular processes [3]. LncRNAs, characterized as RNA molecules longer than 200 nucleotides with limited protein-coding potential, play critical roles in cellular processes through their complex interactions with chromatin structure, transcriptional regulation, and post-transcriptional mechanisms [4]. Recent advancements have further refined the definition of lncRNAs, extending the length criterion to over 500 nucleotides, reflecting an updated understanding of their functional scope and biological significance [5]. Dysregulated expression of lncRNAs in the context of COPD has been intricately linked to processes of inflammation, oxidative stress, and tissue remodeling, underscoring their potential as diagnostic and prognostic markers of disease progression [6].

The intricate interplay between COPD and lung cancer further complicates the clinical landscape, presenting overlapping risk factors such as tobacco smoke exposure and independently amplifying the risk of lung cancer development [7]. This intricate relationship involves a convergence of molecular pathways, creating a microenvironment conducive to malignant transformation [8]. Epidemiological studies indicate that individuals with COPD have a significantly increased risk of developing lung cancer, independent of smoking history. The chronic inflammatory environment in COPD, characterized by persistent oxidative stress and recurrent airway injury, creates a fertile ground for malignant transformation. This inflammatory milieu fosters genomic instability, promotes mutagenesis, and disrupts normal cellular regulatory mechanisms, thereby accelerating the progression from chronic inflammation to carcinogenesis. LncRNAs, which play crucial roles in regulating gene expression, have emerged as key players in both COPD and lung cancer [9]. Dysregulated lncRNAs in COPD influence the expression of tumor suppressors and oncogenes, thereby contributing to the inflammation and cell proliferation that drive the development of lung cancer [10]. By elucidating the specific lncRNA-mediated pathways that connect COPD and lung cancer, we can identify novel biomarkers for early detection and potential therapeutic targets to mitigate the progression from COPD to lung cancer [11]. This integrated approach underscores the importance of molecular research in bridging the gap between chronic inflammatory diseases and cancer, offering new avenues for intervention and improved patient outcomes.

The present review embarks on a comprehensive overview of COPD’s multifaceted background, accentuating clinical challenges and the pressing need for deeper molecular insights. Central to this discourse is the pivotal role of lncRNAs in COPD pathophysiology and their potential as both biomarkers and modulators of key signaling cascades for disease progression. Moreover, this review summarizes the intricate linkages between COPD and lung cancer progression, shedding light on common molecular signatures and their potential therapeutic interventions.

## 2. Chronological Progression of COPD

COPD is a complex and multifactorial respiratory condition characterized by persistent airflow limitation and respiratory symptoms, including cough, sputum production, and dyspnea [12]. Its progression often takes several decades and is marked by distinct stages, each contributing to the gradual deterioration of respiratory function [13]. This review delves into the mode and mechanism of the progression of COPD and explores current approaches to its diagnosis and monitoring.

### 2.1. Disease Progression in COPD

The pathogenesis of COPD comprises various stages, involving both structural and functional changes to the respiratory system. It typically occurs after prolonged exposure to noxious substances, such as cigarette smoke, that cause chronic inflammation, mucus hypersecretion, and alterations in ciliary function [14]. This initial insult triggers a cascade of events, including oxidative stress and protease–antiprotease imbalances, resulting in the destruction of lung parenchyma and shortness of breath (emphysema) [15].

As COPD advances, the airway is remodeled by elevating smooth muscle mass, fibrosis, and narrowing lumens. Chronic bronchitis may manifest with persistent cough and sputum production. The lung function, measured as the forced expiratory volume in one second (FEV1), gradually declines, marking a critical juncture of the disease progression [16]. In later stages, respiratory functions may deteriorate, contributing to worsened disease morbidity and mortality [17]. Furthermore, COPD-associated cachexia, manifested as weight loss and muscle dysfunction, exacerbates the overall impact of the disease [18]. Understanding the disease progression, as well as identification of individuals at risk and the mitigation of exposure and inflammation, is essential for COPD management and prevention [19].

### 2.2. The Major Causative Factors of COPD

The development and progression of COPD are influenced by a myriad of factors, including genetic predisposition, environmental exposures, and individual lifestyle behaviors [20]. Understanding the interplay between these factors is essential for elucidating the pathogenesis of COPD and developing effective prevention and management strategies (Supplementary Figure S1). We will introduce several major causative factors of COPD below.

Air Pollution: Outdoor and indoor air pollution represent significant risk factors for COPD [21]. Inhalation of fine particulate matter, including nitrogen dioxide, sulfur dioxide, ozone from vehicle emissions, industrial activities, and biomass combustion, increases the risk of respiratory symptoms such as COPD. Urbanization, industrialization, and climate change exacerbate air pollution levels, raising public concerns about COPD [22]. Implementation of clean air policies, sustainable transportation strategies, and new energy sources is critical for reducing air pollution and protecting respiratory health [23].

Environmental Exposures: Exposure to environmental pollutants, occupational hazards, and indoor air contaminants contributes to the development and exacerbation of COPD [24]. Inhalation of particulate matter, chemical fumes, and noxious gases from industrial processes, biomass burning, and indoor cooking fuels leads to airway inflammation, oxidative stress, and lung damage in susceptible individuals. Environmental regulations, workplace safety measures, and indoor air quality improvements are essential for reducing environmental exposures and mitigating the burden of COPD in the general population [25].

Cigarette Smoking: Cigarette smoking stands as the primary causative factor for the development of COPD. The inhalation of toxic substances present in cigarette smoke, such as tar, nicotine, and carbon monoxide, triggers inflammation and damage to the airways and lung parenchyma [26]. Chronic exposure to cigarette smoke leads to the progressive destruction of lung tissue, airflow limitation, and respiratory symptoms characteristic of COPD. Moreover, smoking cessation remains the most effective intervention to slow disease progression and reduce morbidity and mortality associated with COPD [27].

Gender: Gender differences play a significant role in the prevalence and clinical manifestations of COPD [28]. Historically, COPD has been more prevalent among males due to higher rates of smoking. However, recent studies have shown an increasing burden of COPD among females, reflecting changes in smoking behaviors as well as the occurrence of environmental irritants that preferentially afflict women [29].

Differences in Smoking Patterns: Historically, COPD has been more strongly associated with males due to higher rates of smoking among men. However, smoking patterns have changed over time, with an increasing number of females smoking cigarettes. In recent years, smoking prevalence among females has risen, contributing to a higher incidence of COPD in women [30].

Biological Differences: Biological factors play a significant role in the development and progression of COPD. Generally, females have narrower airways compared to males, which may contribute to increased susceptibility to COPD. Additionally, hormonal differences between males and females, such as estrogen levels, may influence inflammatory responses to pollutants [31].

Exposure to Indoor Air Pollutants: Women may be more exposed to indoor air pollutants such as biomass fuel smoke, cooking fumes, and secondhand smoke, particularly in regions where indoor cooking with solid fuels is common. Prolonged exposure to these pollutants can increase the risk of developing COPD [32].

Occupational Exposures: Certain occupational exposures, such as working in industries with high levels of airborne pollutants or dust, may disproportionately affect females and contribute to the development of COPD. For example, women working in women-dominant occupations such as house cleaning and textile manufacturing may be exposed to respiratory hazards that increase their risk of COPD [33].

Socioeconomic Factors: Socioeconomic factors, including income level, education, and access to healthcare, influence the risk of COPD development and disease outcomes. Individuals from lower socioeconomic backgrounds are disproportionately affected by COPD due to higher rates of smoking, occupational exposures, and limited access to healthcare services [34,35,36,37]. Socioeconomic disparities contribute to delayed diagnosis, inadequate treatment, and poorer prognosis in COPD patients from disadvantaged communities [38]. Addressing socioeconomic inequalities is essential for reducing the burden of COPD among vulnerable populations [39].

Aging: Aging is a major risk factor for the development of COPD. Age-related lung structure and function changes, including decreased lung elasticity, reduced mucociliary clearance, and impaired immune responses, predispose older adults to COPD and exacerbations [40]. Moreover, cumulative exposure to environmental pollutants, respiratory infections, and other comorbidities further exacerbates the decline of lung functions and disease progression in elderly individuals. Comprehensive geriatric assessment and management strategies are essential for improving COPD care and outcomes in older patients [41].

Respiratory Infections: Respiratory infections, particularly viral and bacterial infections, contribute to the pathogenesis and exacerbations of COPD. Acute respiratory infections, such as influenza and pneumonia, trigger airway inflammation, mucous production, and exacerbation in COPD patients [42]. Moreover, recurrent respiratory infections accelerate the decline of lung functions and worsen the clinical outcomes of COPD patients. Vaccination and appropriate antimicrobial therapies play crucial roles in preventing and managing COPD [43].

Genetic Factors: Genetic predisposition plays a significant role in COPD susceptibility and disease heterogeneity [44]. Variations in genes involved in lung development, inflammation, and immunological defense influence susceptibility to COPD and response to environmental exposures, such as cigarette smoke and air pollution [45]. For example, alpha-1 antitrypsin deficiency, an inherited disorder, is a well-established genetic risk factor for early-onset COPD. Understanding the genetic basis of COPD facilitates early identification of at-risk individuals and personalized treatment based on genetic profiling [46].

### 2.3. Diagnosis and Monitoring of COPD

Accurate and timely diagnosis of COPD is fundamental for effective management and intervention. Diagnosis is typically established through a combination of clinical assessment, spirometry, and consideration of risk factors such as smoking history and environmental exposures [47]. Spirometry, measuring the FEV1 and the ratio of FEV1 to forced vital capacity (FVC), remains the gold standard for diagnosing airflow limitation [48]. Monitoring disease progression involves regular assessments of symptoms, exacerbation history, and lung function. The global initiative for chronic obstructive lung disease (GOLD) classification provides a framework for categorizing disease severity based on spirometric measurements [49]. However, it is increasingly recognized that a comprehensive assessment of COPD should extend beyond spirometry, encompassing symptoms, exacerbation risk, and impact on patient health status [50]. Novel approaches to COPD monitoring involve the exploration of biomarkers and imaging techniques to provide a more nuanced understanding of disease activity. Blood biomarkers, such as C-reactive protein (CRP) and fibrinogen, have shown promise in reflecting systemic inflammation and predicting exacerbation risk [51]. Imaging modalities, including computed tomography (CT) and magnetic resonance imaging (MRI), offer insights into structural lung changes and phenotypic variations, aiding in personalized therapeutic strategies [52]. While traditional diagnostic and monitoring methods, including spirometry, clinical assessment, and imaging, remain indispensable in the management of COPD, emerging molecular approaches offer the potential for more precise and personalized care. Among these, lncRNAs have gained attention as novel biomarkers that can provide deeper insights into the pathophysiological mechanisms of COPD [53]. By integrating lncRNA profiling into clinical practice, we can enhance our ability to predict disease progression, tailor therapeutic interventions, and improve patient outcomes. This molecular dimension adds a significant layer of specificity and sensitivity to COPD diagnosis and monitoring, paving the way for innovative strategies in the fight against this debilitating disease [54]. The following section delves into the prognostic and diagnostic significance of lncRNAs in COPD, highlighting their potential to revolutionize our understanding and management of the disease.

## 3. Exploring LncRNA Profiles and Clinical Significance in COPD

COPD is a multifaceted respiratory condition characterized by persistent airflow limitation and chronic inflammation [55]. Recent advances in molecular biology have highlighted the pivotal role of lncRNAs in the pathogenesis and progression of COPD. Exploring lncRNA profiles in COPD patients has revealed distinct expression patterns associated with disease severity, exacerbation frequency, and response to therapy [56]. These lncRNAs, which were once considered transcriptional noise, are now recognized as critical regulators of gene expression, influencing inflammatory pathways, immune responses, and cellular processes such as apoptosis and proliferation [57]. By mapping the lncRNA landscape in COPD, researchers have identified potential biomarkers for early diagnosis, disease monitoring, and personalized treatment strategies. The clinical significance of lncRNAs extends beyond their biomarker potential; they also offer new therapeutic targets, providing a novel avenue for intervention in COPD management [58]. Understanding the intricate roles of lncRNAs in COPD not only enhances our comprehension of the disease’s molecular underpinnings but also opens up innovative pathways for improving patient outcomes.

### 3.1. Identification and Profiling of COPD-Associated LncRNAs

**Figure 1:**
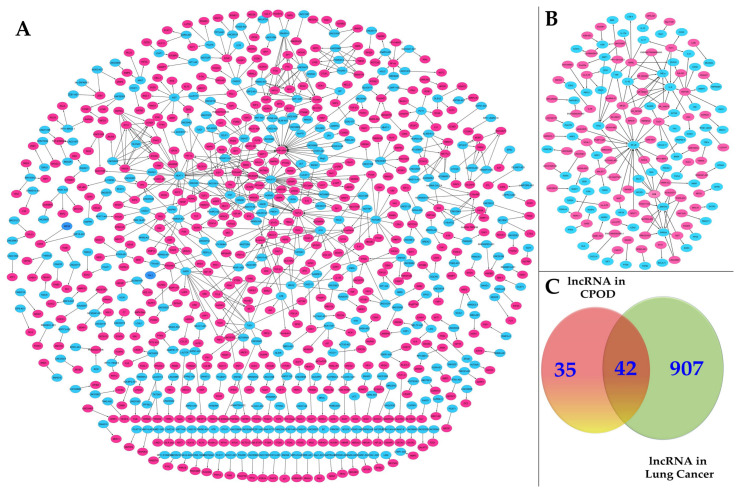
Profiling of LncRNAs in COPD and lung cancer. (**A**) Representative network of interacting lncRNAs/mRNAs in lung cancer: This network visualization offers a comprehensive view of the intricate interactions between these RNA species (indirect interaction mediated by miRNAs), highlighting their regulatory roles and potential significance in the context of lung cancer. By mapping out these interactions, this representation aims to unravel the complex molecular mechanisms underlying lung cancer development and progression. (**B**) Representative network of interacting lncRNAs/mRNAs in COPD: This network elucidates the intricate relationships between these RNA molecules, shedding light on their regulatory roles and potential implications in COPD pathogenesis. Through systematic analysis, this representation aims to provide insights into the underlying molecular mechanisms driving COPD progression. (**C**) Venn Diagram to illustrate the shared lnRNAs between COPD and lung cancer: This visual representation showcases the overlapping lncRNA profiles between the two respiratory conditions, offering valuable insights into potential common molecular pathways and regulatory mechanisms; the blue color indicates lncRNAs and the red color indicates mRNAs. This figure created using Cytoscape 3.10.2 software, integrates data derived from Supplementary Excel Files compiled from the PubMed database.

The validated lncRNAs associated with COPD and lung cancer have been extensively reviewed in the current literature (Figure 1). These lncRNAs, in conjunction with miRNAs, play a significant role in interacting with mRNAs, thereby influencing the progression of COPD and/or cancer through competitive endogenous RNA (ceRNA) networks (Supplementary Excel Files S1 and S2). The ceRNA hypothesis posits that lncRNAs can function as molecular sponges for miRNAs, thereby modulating the expression of miRNA target genes [59]. This complex interaction framework highlights the potential of lncRNAs to impact COPD (Supplementary Excel File S1) and lung cancer (Supplementary Excel File S2). By acting as ceRNAs, lncRNAs can sequester miRNAs, preventing them from binding to their target mRNAs, which in turn affects gene expression and cellular processes involved in disease progression [60]. This regulatory mechanism provides a new perspective on the roles of lncRNAs in the pathogenesis of COPD and lung cancer, opening up new research directions and offering insights into the molecular underpinnings of these diseases.

Identifying and profiling COPD-associated lncRNAs represents a critical step toward understanding the molecular mechanisms underlying the disease’s pathogenesis and progression. Genome-wide expression profiling studies have consistently revealed dysregulated expression patterns of lncRNAs in COPD patients compared to healthy controls, underscoring their potential utility as diagnostic and prognostic biomarkers [61]. Such dysregulation highlights the importance of lncRNAs in the disease process and their potential role in the development of therapeutic strategies.

Recent advancements in high-throughput sequencing technologies and bioinformatics tools have facilitated the systematic characterization of lncRNA expression profiles in COPD [62]. Transcriptomic analyses utilizing next-generation sequencing platforms have enabled the comprehensive identification of novel lncRNAs and the elucidation of their regulatory networks in COPD pathophysiology [63].

One of the challenges in lncRNA profiling studies is the heterogeneity of COPD phenotypes and disease severity, which necessitates careful selection of patient cohorts and appropriate control groups for comparative analyses [64]. Integrative approaches combining transcriptomic data with clinical parameters, imaging findings, and functional assays are essential for prioritizing candidate lncRNAs and elucidating their biological significance in COPD [65]. A thorough understanding of the regulatory mechanisms controlling lncRNA expression in COPD is essential for elucidating their functional roles and clinical significance. Emerging evidence indicates that lncRNAs undergo dynamic regulation by transcription factors, epigenetic modifiers, and signaling pathways involved in COPD pathogenesis. Moreover, these lncRNAs influence mRNA levels, a process frequently mediated by miRNAs through competitive endogenous RNA interactions [66].

### 3.2. Master Regulators of Gene Expression in Lung Cancer Progression

**Figure 2:**
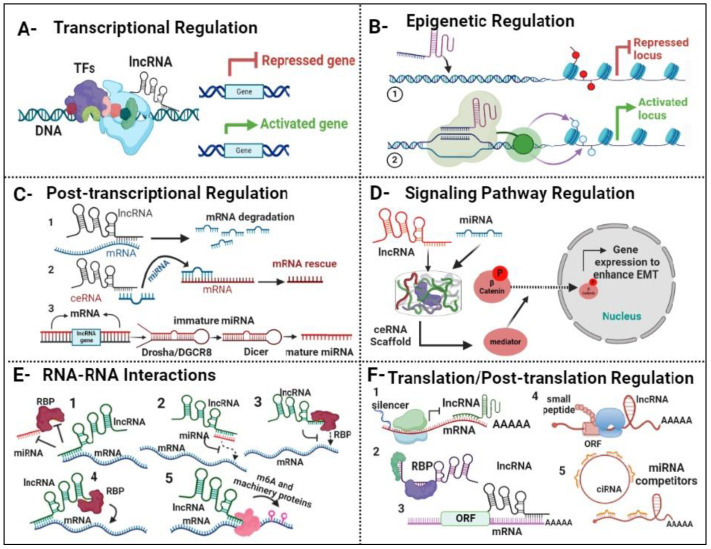
LncRNA regulators of gene expression in lung cancer. (**A**) Transcriptional regulation: LncRNA interacts either with transcription factors (TFs) or directly with DNA, resulting in either repression or enhancement of gene expression. (**B**) Epigenetic Regulation by LncRNAs. lncRNAs mediate the repression of target genes through modifications to histone proteins, leading to altered chromatin states that silence gene expression (1), lncRNAs facilitate the activation of target genes by modifying histone proteins, promoting a chromatin environment that supports transcriptional activity (2). (**C**) Post-transcriptional regulation: LncRNA directly binds to mRNA, leading to its degradation (1); lncRNA can also act as a competitive inhibitor with miRNA, forming a scaffold that binds to mRNA, thereby rescuing it from degradation (2). Additionally, lncRNA plays a role in facilitating the maturation process of miRNA (3). (**D**) Signaling pathway regulation: LncRNA and miRNAs collaborate to create a scaffold complex, orchestrating the beta-catenin signaling pathway. This complex regulates gene expression to promote the epithelial–mesenchymal transition (EMT) process. (**E**) RNA-RNA interaction: LncRNAs play a pivotal role in modulating mRNA stability through various mechanisms. These include sequestering miRNAs or RNA-binding proteins (RBPs) to prevent their interaction with mRNA molecules (1), directly binding to miRNAs (2) or RBPs (3), and binding to mRNA via RBPs (4). Additionally, lncRNA can regulate RNA modification by interacting with m6A machinery proteins (5). (**F**) Translation/post-translation regulation: cytoplasmic lncRNA compete for ribosomal protein to mRNA to regulate expression by regulating mRNA stability (1); lncRNA binds to RBPs (2); mRNA translation (3); few lncRNA contain small open reading frames (ORFs) that can be translated in biological active small peptides (4). In addition, lncRNA competes for miRNA binding (5). The figure was designed using the BioRender platform based on reference sources [80,81,82].

LncRNAs are pivotal regulators of gene expression in lung cancer progression. They control gene expression through various mechanisms, including epigenetic modifications, competition with microRNAs (miRNAs), and direct interaction with mRNA transcripts [67]. Through these diverse pathways, lncRNAs exert precise control over gene expression programs implicated in lung cancer progression (Figure 2), underscoring their importance in the disease’s molecular landscape [68].

*Transcriptional Regulation:* LncRNAs are known to play crucial roles in transcriptional regulation by influencing gene expression at the chromatin level [69]. One such example is MALAT1, which has been extensively studied in the context of lung cancer metastasis. MALAT1 functions as a key regulator of gene transcription by interacting with transcription factors and chromatin-modifying proteins [70]. Through these interactions, MALAT1 modulates the expression of genes involved in various aspects of lung cancer progression, including metastasis. For instance, MALAT1 has been shown to promote the expression of metastasis-associated genes by facilitating chromatin remodeling and transcriptional activation [71].

*Epigenetic Regulation:* Epigenetic regulation mediated by lncRNAs is a fundamental mechanism governing gene expression in lung diseases. An illustrative example is HOTAIR, which has been implicated in epigenetic modifications associated with lung cancer progression [72]. HOTAIR functions as a scaffold for chromatin-modifying complexes, such as polycomb repressive complex 2 (PRC2), leading to alterations in histone methylation patterns. Specifically, HOTAIR interacts with PRC2 to promote the deposition of repressive histone marks, such as histone H3 lysine 27 trimethylation (H3K27me3), at target gene promoters [73].

*Post-transcriptional Regulation:* Post-transcriptional regulation by lncRNAs plays a critical role in modulating gene expression levels and mRNA processing in lung diseases. A notable example is GAS5, which functions as a competing endogenous RNA (ceRNA) in idiopathic pulmonary fibrosis (IPF) [74]. GAS5 has been shown to sequester miR-21, a known regulator of fibrosis-related genes, thereby relieving its inhibitory effect on target mRNAs. By acting as a molecular sponge for miR-21, GAS5 promotes the expression of its target gene PTEN (Phosphatase and Tensin Homolog), leading to decreased cell proliferation and fibrosis progression in IPF [75].

*Signaling Pathway Regulation:* LncRNAs are involved in the regulation of signaling pathways that govern cellular processes in lung diseases, offering potential targets for therapeutic intervention. An exemplary lncRNA in this context is H19, which modulates the transforming growth factor-beta (TGF-β) signaling pathway in lung cancer [76]. H19 has been shown to enhance TGF-β signaling by promoting the phosphorylation and nuclear translocation of Smad2/3, key mediators of the TGF-β pathway. This enhanced signaling cascade induces epithelial–mesenchymal transition (EMT) and promotes metastasis in lung cancer cells [77].

*RNA-RNA Interactions:* Interactions between lncRNAs and other RNA molecules contribute to the complex regulatory networks governing gene expression in lung diseases. A notable example is the lncRNA XIST, which interacts with the mRNA of the tumor suppressor gene RBM5 (RNA Binding Motif Protein 5) in lung cancer cells [78]. XIST-mediated sequestration of RBM5 mRNA leads to decreased protein expression levels, resulting in enhanced proliferation and survival of lung cancer cells [79]. This RNA-RNA interaction exemplifies the intricate crosstalk between lncRNAs and mRNAs, highlighting their role in shaping the molecular landscape of lung diseases.

### 3.3. Tobacco Smoke Exposure and Expression Profiles of LncRNA in COPD

**Figure 3:**
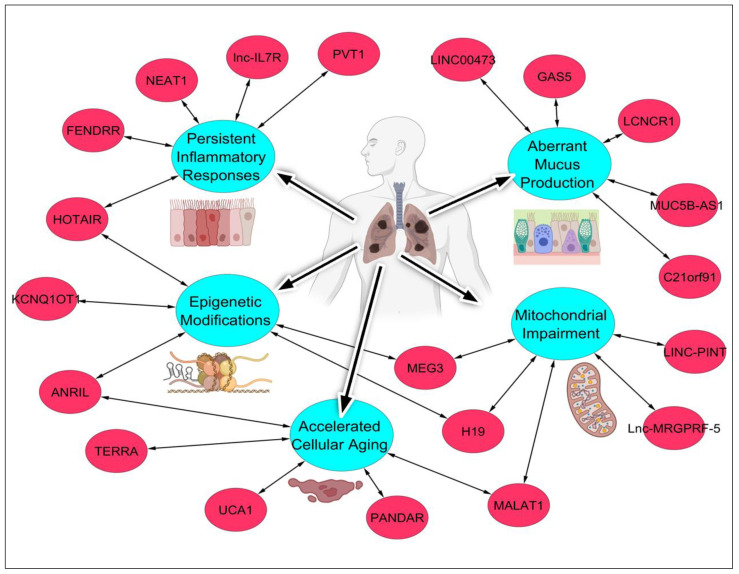
Overview highlighting the role of tobacco smoke in modulating LncRNAs during COPD pathogenesis. Exposure to tobacco smoke induces significant alterations in the expression and functions of lncRNAs, precipitating a cascade of augmented COPD pathologies characterized by mitochondrial impairment, persistent inflammatory response, aberrant mucus production, epigenetic modifications, and cellular senescence or accelerated cellular aging. Noteworthy lncRNAs implicated in this multifaceted process include ANRIL, C21orf91, FENDRR, GAS5, H19, HOTAIR, KCNQ1OT1, LCNCR1, LINC00473, LINC-PINT, lnc-IL7R, Lnc-MRGPRF-5, MALAT1, MEG3, MUC5B-AS1, NEAT1, PANDAR, PVT1, TERRA, and UCA1, each intricately woven into the intricate tapestry of COPD-associated molecular dysregulation. The figure was designed using the BioRender platform, and incorporates information derived from reference sources [118], and from the PubMed database on ANRIL, C21orf91, FENDRR, GAS5, H19, HOTAIR, KCNQ1OT1, LCNCR1, LINC00473, LINC-PINT, lnc-IL7R, Lnc-MRGPRF-5, MALAT1, MEG3, MUC5B-AS1, NEAT1, PANDAR, PVT1, TERRA, and UCA1.

Exposure to tobacco smoke is a major risk factor for the development and progression of COPD, a complex and debilitating respiratory condition characterized by chronic inflammation, airway obstruction, and irreversible lung damage (Figure 3) [83]. Tobacco smoke contains thousands of toxic compounds, including carcinogens and reactive oxygen species, which can induce widespread genomic alterations and dysregulate various cellular processes in the lung microenvironment [84]. Emerging evidence suggests that exposure to tobacco smoke profoundly influences the landscape of lncRNAs in COPD, contributing to disease pathogenesis and progression. This reflects the complex interplay between environmental stimuli, genetic predisposition, and epigenetic modifications in disease pathophysiology [85]. High-throughput sequencing technologies and genome-wide expression profiling have facilitated the identification of tobacco smoke-responsive lncRNAs associated with COPD susceptibility, severity, and treatment outcomes. These dysregulated lncRNAs exhibit altered expression patterns in response to tobacco smoke exposure, modulating key cellular processes involved in inflammation, oxidative stress, and tissue remodeling in the lung [86].

Functional characterization of tobacco smoke-responsive lncRNAs provides insights into their roles as critical regulators of gene expression networks and signaling pathways implicated in COPD pathogenesis [87]. Tobacco smoke-induced lncRNAs, such as HOTAIR, MALAT1, and NEAT1, modulate inflammatory responses and fibrotic processes by interacting with chromatin-modifying complexes, transcription factors, and RNA-binding proteins [88]. Dysregulated expression of these lncRNAs contributes to exacerbated airway inflammation, oxidative stress, and tissue damage in COPD patients [89]. Furthermore, tobacco smoke exposure alters the epigenetic landscape of the lung, leading to aberrant DNA methylation patterns, histone modifications, and chromatin remodeling events that regulate lncRNAs’ expression and function in COPD [90]. Such epigenetic changes may reprogram the transcriptional profiles of lncRNAs involved in immune response, cell proliferation, and apoptosis, driving disease progression and exacerbation in COPD patients. Understanding the molecular mechanisms for the effects of tobacco smoke exposure on the landscape of lncRNAs in COPD is essential for identifying novel biomarkers and therapeutic targets for disease intervention and personalized treatment approaches [91].

The intricate landscape of lncRNAs has emerged as a crucial player in COPD pathogenesis, orchestrating diverse cellular processes implicated in disease onset and progression. In response to tobacco smoke exposure, aberrant expression patterns of lncRNAs contribute to mitochondrial dysfunction, chronic inflammation, mucus dysregulation, epigenetic alterations, and cellular senescence, which are key pathological hallmarks of COPD [92].

Mitochondrial dysfunction in COPD patients exposed to tobacco smoke is significantly influenced by aberrant lncRNA expression. LINC-PINT, for instance, is downregulated in lung epithelial cells upon exposure to cigarette smoke, resulting in oxidative stress and impaired mitochondrial function [93]. Similarly, H19 has been implicated in mitochondrial dysfunction in lung cells, where its dysregulation exacerbates oxidative damage and respiratory deficiencies [94]. Lnc-MRGPRF-5 is another example, whose overexpression in smokers’ lung tissues is associated with compromised mitochondrial integrity and function [95]. MALAT1, known for its role in various cellular processes, also impacts mitochondrial dynamics and bioenergetics in COPD, with its dysregulation leading to mitochondrial impairment [96]. Lastly, MEG3, reduced in response to cigarette smoke, contributes to mitochondrial dysfunction and oxidative stress, furthering the pathology of COPD [97].

Chronic inflammation is another hallmark of COPD exacerbated by tobacco smoke, with several lncRNAs playing pivotal roles. NEAT1 is notably upregulated in COPD, where it modulates immune responses, contributing to a sustained inflammatory environment [98]. HOTAIR is similarly elevated in COPD patients, promoting inflammatory pathways that exacerbate lung damage due to tobacco exposure [99]. PVT1 overexpression is linked to increased production of inflammatory cytokines, thus perpetuating chronic inflammation in COPD [100]. Lnc-IL7R, involved in immune regulation, is upregulated in smokers’ lung tissues, thereby contributing to the inflammatory milieu [101]. FENDRR, another lncRNA, is dysregulated in COPD, playing a significant role in maintaining chronic inflammation [102].

Mucus dysregulation is a critical feature of COPD, influenced by the aberrant expression of specific lncRNAs. MUC5B-AS1, for instance, regulates the expression of MUC5B, a mucin gene implicated in mucus hypersecretion characteristic of COPD [103]. LCNCR1 is upregulated in response to tobacco smoke, leading to increased mucus production and contributing to airway obstruction in COPD patients [104]. GAS5 influences mucus secretion pathways, and its dysregulation is associated with exacerbated mucus production in COPD [105]. C21orf91 modulates genes involved in mucus regulation, with its dysregulation contributing to the pathological mucus production observed in COPD [106]. Additionally, LINC00473 is implicated in mucus hypersecretion, where its dysregulation exacerbates the mucus-related symptoms of COPD [107].

Epigenetic alterations induced by tobacco smoke are critical in the pathogenesis of COPD, with lncRNAs playing a substantial role. HOTAIR, for example, mediates epigenetic modifications through histone methylation, thus contributing to the disease’s progression [108]. MEG3 affects DNA methylation patterns in lung tissues, with its dysregulation being a significant factor in COPD [109]. ANRIL modulates chromatin remodeling and is implicated in the epigenetic changes associated with COPD [110]. KCNQ1OT1 regulates epigenetic modifications via imprinting control regions, and its dysregulation due to cigarette smoke exposure further complicates COPD pathology [111]. H19 is involved in DNA methylation and histone acetylation, with its aberrant expression contributing to the epigenetic landscape observed in COPD [112].

Cellular senescence is prominently featured in COPD and is exacerbated by tobacco smoke through the dysregulation of lncRNAs. UCA1 is upregulated in COPD, where it contributes to cellular senescence in response to tobacco smoke [113]. MALAT1, another lncRNA, is linked to the promotion of cellular senescence in COPD patients, with its dysregulation exacerbating the disease [114]. TERRA influences telomere maintenance and senescence pathways in lung cells, and its aberrant expression is a significant factor in COPD progression [115]. CDKN2B-AS1 (ANRIL) regulates the expression of senescence-associated genes, with its dysregulation promoting cellular senescence in COPD [116]. Lastly, PANDAR is upregulated in COPD, where it is associated with increased cellular senescence in lung tissues exposed to tobacco smoke [117].

These examples underscore the critical role of lncRNAs in mediating the deleterious effects of tobacco smoke in COPD. Understanding the specific mechanisms by which these lncRNAs contribute to mitochondrial dysfunction, chronic inflammation, mucus dysregulation, epigenetic alterations, and cellular senescence can provide valuable insights into the pathogenesis of COPD and potential therapeutic targets.

### 3.4. Correlation Between LncRNA Expression and COPD Severity

The correlation between lncRNA expression and COPD severity is a topic of significant interest in respiratory medicine, offering insights into disease progression and clinical outcomes [119]. Several studies have reported associations between aberrant lncRNA expression profiles and various parameters of COPD severity, including a decline in lung function, exacerbation frequency, and radiological features [120].

MALAT1, one of the most extensively studied lncRNAs in COPD, has been positively correlated with disease severity metrics such as FEV1 and the BODE index (Body mass index, airflow Obstruction, Dyspnea, and Exercise capacity). Elevated MALAT1 expression levels have been associated with accelerated lung function decline, increased risk of exacerbations, and poorer prognosis in COPD patients [121].

Similarly, HOTAIR expression levels have also been positively correlated with COPD severity scores and radiological evidence of emphysema and airway remodeling. The dysregulation of HOTAIR-mediated signaling pathways, including TGF-β (transforming growth factor-β) and Wnt/β-catenin, has been implicated in the pathogenesis of COPD and the progression of emphysema [122]. The prognostic value of lncRNAs in predicting COPD outcomes extends beyond their individual expression levels to encompass their interactions with other molecular mediators and cellular pathways. Integrative analyses incorporating lncRNA signatures with other clinical predictors and biomarkers of COPD hold promise for refining risk stratification models and optimizing therapeutic strategies for the disease [123].

### 3.5. Prognostic and Diagnostic Value of LncRNA in Predicting COPD Outcomes

LncRNAs have emerged as crucial regulators of gene expression, exerting significant influence on various biological processes. Unlike their protein-coding counterparts, lncRNAs do not encode proteins but instead orchestrate diverse cellular functions through mechanisms such as chromatin remodeling, transcriptional regulation, and post-transcriptional processing [124]. In the context of COPD, the dysregulation of lncRNAs has been implicated in disease pathogenesis and progression, offering promising avenues for prognostic and diagnostic applications. COPD is a multifactorial respiratory disorder characterized by persistent airflow limitation and progressive decline in lung function [125]. Despite advances in therapeutic strategies, COPD remains a leading cause of morbidity and mortality worldwide [126]. Therefore, the identification of reliable prognostic and diagnostic biomarkers is imperative for early disease detection, prognostic assessment, and personalized treatment strategies.

LncRNAs are particularly noteworthy due to their tissue-specific expression patterns, stability in various biological fluids, and functional relevance in COPD pathophysiology [127]. Certain lncRNAs may be predominantly expressed in specific cell types within the lung, such as epithelial cells, fibroblasts, or immune cells, which can significantly influence their functional roles and impact disease mechanisms [128]. Certain lncRNAs may be predominantly expressed in specific cell types within the lung, such as epithelial cells, fibroblasts, or immune cells, which can significantly influence their functional roles and impact disease mechanisms. This cell-type specificity can significantly impact their role as biomarkers and therapeutic targets. LncRNAs are particularly noteworthy due to their tissue-specific expression patterns, stability in various biological fluids, and functional relevance in COPD pathophysiology [129]. For instance, HOTAIR is highly expressed in lung epithelial cells and has been implicated in lung cancer progression by modulating chromatin states and gene expression [130]. In fibroblasts, MALAT1 has been shown to regulate fibroblast activation and fibrosis, contributing to COPD pathogenesis [131]. Immune cell-specific lncRNAs such as NEAT1 play a role in modulating immune responses and inflammation, which are critical in both COPD and lung cancer [132]. Understanding these cell-type-specific expression profiles is crucial as they can significantly influence the functional roles of lncRNAs and their impact on disease mechanisms.

The prognostic value of lncRNAs in predicting COPD outcomes is a burgeoning area of research, with implications for disease monitoring, treatment response prediction, and personalized patient care [133]. Longitudinal cohort studies have identified specific lncRNA signatures associated with disease progression, exacerbation risk, and mortality in COPD patients. GAS5, a well-characterized lncRNA involved in the regulation of apoptosis, has emerged as a good prognostic biomarker for COPD outcomes [134]. Decreased GAS5 expression levels are correlated with increased risk of exacerbations, hospitalizations, and mortality among COPD patients [135]. Conversely, NEAT1, a nuclear-enriched lncRNA involved in the regulation of chromatin organization and gene expression, has been linked to a worse prognosis in COPD patients [136]. Elevated NEAT1 expression levels are associated with an accelerated decline in lung function, poorer response to bronchodilator therapy, and increased mortality. Similarly, elevated expression levels of MALAT1 and NEAT1 are correlated with adverse clinical outcomes and poorer prognosis [137]; these observations strongly suggest that these lncRNAs serve as prognostic indicators for risk stratification and treatment optimization, as well as diagnostic biomarkers for COPD, offering potential alternatives or complementary tools to traditional clinical assessments [138]. Furthermore, the expression levels of certain lncRNAs such as MALAT1, HOTAIR, and GAS5 predict COPD phenotypes and the degrees of disease severity and exacerbation risk [139].

Beyond their diagnostic and prognostic implications, lncRNAs also hold promise as therapeutic targets and predictors of disease progression and therapeutic response for COPD, enabling personalized treatment regimens tailored to individual patient profiles [140]. The dysregulation of lncRNAs contributes to key pathophysiological processes underlying COPD, including inflammation, oxidative stress, and airway remodeling. Targeted modulation of such dysregulated lncRNAs through pharmacological interventions or gene therapy represents a promising strategy for mitigating disease progression and improving patient outcomes [141].

## 4. Regulatory Functions of LncRNAs in COPD

LncRNAs regulate gene expression through various mechanisms at different stages of gene expression, including transcription, RNA processing, and translation. These mechanisms involve interactions with chromatin-modifying complexes, transcription factors, miRNAs, and other RNA-binding proteins [142].

*Chromatin Remodeling:* LncRNAs can influence chromatin structure and accessibility by recruiting chromatin-modifying complexes to specific genomic loci. For example, lncRNAs can interact with histone-modifying enzymes such as histone methyltransferases or histone deacetylases to promote or inhibit histone modifications that regulate gene expression [143]. By altering chromatin structure, lncRNAs can control the accessibility of transcriptional machinery to target genes [144].

*Transcriptional Regulation:* LncRNAs can directly influence transcriptional activity by interacting with transcription factors or RNA polymerase complexes. Some lncRNAs act as co-activators or co-repressors of transcription by binding to transcription factors and modulating their activity or recruitment to target gene promoters [145]. Others serve as scaffolds for the assembly of transcriptional complexes, bringing together regulatory proteins and DNA elements to regulate gene expression [146].

*RNA Processing and Stability:* LncRNAs can regulate RNA processing events, such as alternative splicing, RNA editing, and RNA stability. By binding to pre-mRNA transcripts or RNA processing factors, lncRNAs can influence splice site selection or exon inclusion/exclusion patterns, leading to the production of different mRNA isoforms with distinct functions [147]. Additionally, lncRNAs can stabilize or destabilize target mRNAs by forming RNA duplexes or competing for binding with miRNAs, thereby modulating mRNA turnover and translation efficiency [148].

*Epigenetic Regulation:* LncRNAs can regulate gene expression in a heritable manner by influencing epigenetic modifications such as DNA methylation and histone modification patterns. Some lncRNAs act as guides or scaffolds for recruiting chromatin-modifying enzymes to specific genomic loci, leading to changes in DNA methylation status or histone acetylation/methylation patterns that affect gene expression. These epigenetic changes can be stably inherited through cell divisions and play important roles in cell fate determination and differentiation [149].

*Subcellular Localization:* LncRNAs can localize to specific subcellular compartments, such as the nucleus or cytoplasm, where they exert distinct regulatory functions [150]. Nuclear lncRNAs often regulate transcriptional processes by interacting with chromatin or transcriptional machinery, while cytoplasmic lncRNAs may modulate post-transcriptional events such as mRNA stability, translation, or protein localization. Overall, lncRNAs play diverse and complex roles in the regulation of gene expression, acting at multiple levels to fine-tune cellular processes and maintain cellular homeostasis [151].

Given the multifaceted roles of lncRNAs in regulating gene expression and maintaining cellular homeostasis, it is evident that they also play crucial roles in the immune dysregulation observed in COPD. By influencing key molecular pathways and cellular processes, lncRNAs contribute to the chronic inflammation and immune responses characteristic of COPD [152]. This regulatory influence is particularly significant in the modulation of immune-related genes and signaling pathways, which underscores the importance of understanding lncRNA-mediated immune responses in the pathogenesis of COPD. The next section will delve into the specific lncRNAs involved in these immune mechanisms and their impacts on COPD progression.

### 4.1. LncRNA-Mediated Immune Response in COPD

COPD is characterized by persistent inflammation and immune dysregulation in the airways and lung parenchyma, driven by complex interactions between immune cells, cytokines, and regulatory molecules. LncRNAs have emerged as critical regulators of the immune response in COPD, modulating inflammatory signaling pathways, immune cell differentiation, and cytokine production in the lung microenvironment [153]. Several lncRNAs, including NEAT1 [154], GAS5 [155], MALAT1 [156], HOTAIR [157], and TUG1 [158], have been implicated in the modulation of immune responses in COPD. These lncRNAs interact with RNA-binding proteins, miRNAs, and transcription factors to regulate the expression of immune-related genes and signaling pathways involved in COPD pathogenesis [159].

NEAT1 is a nuclear retained lncRNA that forms the structural scaffold for the formation of nuclear bodies known as paraspeckles. In COPD, dysregulated expression of NEAT1 alters inflammatory cytokine production and immune cell function, contributing to disease progression [160]. NEAT1 interacts with RNA-binding proteins like SFPQ and transcription factors such as RELA to regulate the expression of immune-related genes involved in COPD pathogenesis [161].

GAS5 is a stress-induced lncRNA that regulates the proliferation and apoptosis of immune cells, including T cells and macrophages. Dysregulated expression of GAS5 in COPD affects immune cell function and inflammatory cytokine production, exacerbating airway inflammation and tissue damage [162]. GAS5 modulates the activity of key signaling pathways involved in COPD pathogenesis, including NF-κB and JAK-STAT signaling, by binding to the glucocorticoid receptor and sequestering miRNAs such as miR-21, which in turn modulates the expression of target genes [163].

HOTAIR is an oncogenic lncRNA implicated in the regulation of immune responses in COPD. Dysregulated expression of HOTAIR alters the expression of immune-related genes and cytokines, contributing to chronic inflammation and tissue damage in COPD patients [164]. HOTAIR interacts with chromatin-modifying complexes such as PRC2 and LSD1 and transcription factors like STAT3 to regulate the expression of genes involved in immune cell activation and cytokine signaling [165].

MALAT1 is a highly conserved lncRNA that regulates immune responses and inflammatory signaling pathways in COPD. Dysregulated expression of MALAT1 affects immune cell infiltration, cytokine production, and tissue remodeling processes in COPD patients [166]. MALAT1 interacts with RNA-binding proteins such as HuR and miRNAs like miR-146a, modulating the expression of genes involved in inflammatory responses and immune cell function [167].

TUG1 is an lncRNA implicated in the regulation of immune responses and inflammatory signaling pathways in COPD. Dysregulated expression of TUG1 alters the expression of immune-related genes and cytokines, contributing to airway inflammation and tissue remodeling in COPD patients [168]. TUG1 interacts with chromatin-modifying complexes such as EZH2 and transcription factors like NF-κB to regulate the expression of genes involved in immune cell activation and inflammatory responses [169].

### 4.2. Complexities of LncRNA-Mediated Inflammation in COPD

**Figure 4:**
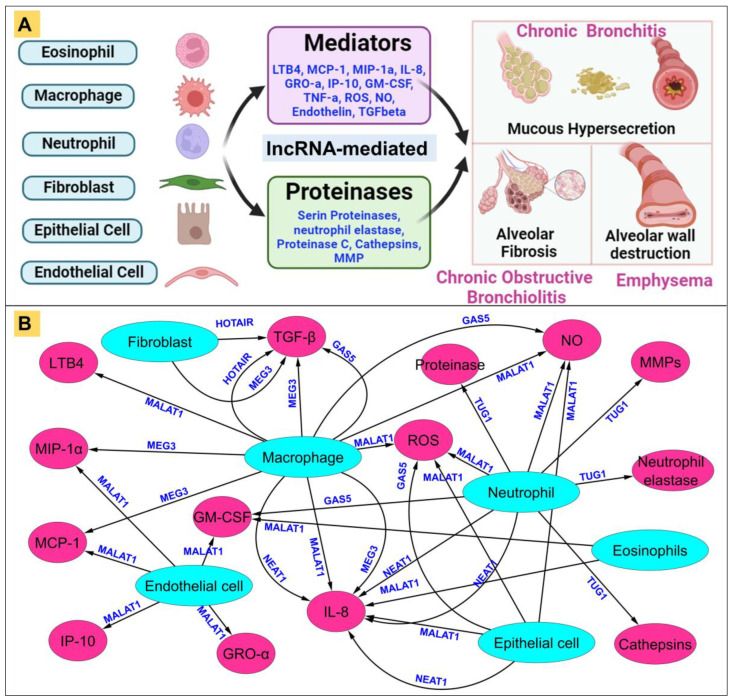
The multifaceted landscape of inflammation in COPD: (**A**) Inflammation complexity in COPD. The intricate tapestry of inflammation in COPD unfurls with myriad activated inflammatory and structural cells, orchestrating a symphony of mediators. Among them, lipid mediators like LTB4 beckon neutrophils, while chemokines such as MCP-1 and MIP-1α allure monocytes. IL-8 and GRO-α extend their call to both neutrophils and monocytes, while IP-10 beckons CD8+ cells alongside ROS and NO. GM-CSF prolongs the survival of neutrophils, while TNF-α ignites inflammation by activating multiple inflammatory genes and may underscore systemic manifestations of the disease. Meanwhile, endothelin and TGF-β set the stage for fibrosis. Concurrently, a host of proteinases including neutrophil elastase, proteinase C, cathepsins, and MMPs orchestrate elastolysis, ushering in the hallmark pathophysiology of COPD characterized by inflammatory cell activation, elastolysis, and mucus hypersecretion. (**B**) Representative network of epithelial and immune cell contributions in COPD: Epithelial and immune cells, including neutrophils, macrophages, and eosinophils, play a crucial role in COPD by secreting proteinases and mediators that drive inflammation. NEAT1, MALAT1, HOTAIR, MEG3, GAS5, and TUG1 are key lncRNAs regulating this process. NEAT1 and MALAT1 modulate IL-8 production in epithelial cells, neutrophils, and macrophages, while MALAT1 also affects ROS, NO, and several chemokines in endothelial cells. HOTAIR influences TGF-β and TNF-α in fibroblasts and macrophages, and MEG3 regulates TGF-β, MCP-1, MIP-1α, and IL-8 in macrophages. GAS5 affects ROS, NO, GM-CSF, and TNF-α in various cells, and TUG1 controls neutrophil elastase and other proteases. These interactions exacerbate the inflammatory milieu in the airways, highlighting the potential of lncRNAs as therapeutic targets for COPD. (**A**) was designed using BioRender, incorporating data from reference sources [196]. (**B**), developed with Cytoscape software, is based on additional references from PubMed.

Recent studies have implicated lncRNAs as key regulators of inflammatory pathways in various diseases, including COPD [170]. Dysregulated expression of lncRNAs has been associated with aberrant inflammatory responses, airway remodeling, and disease progression. Several lncRNAs have been identified as critical regulators of inflammatory signaling pathways in COPD pathogenesis, modulating the expression of key mediators involved in COPD-associated inflammation, such as chemokines, growth factors, and proteases [171]. The contributing epithelial cells (endothelial, fibroblast, and epithelial cells) and immune cells (neutrophils, macrophages, and eosinophils) secrete mediators and proteinases that orchestrate the recruitment and activation of inflammatory cells, intensifying the inflammatory environment within the airways through the regulation of lncRNAs (Figure 4A) [172].

In the pathogenesis of COPD, lncRNAs play pivotal roles by modulating the activities of various immune and structural cells, including fibroblasts, CD8+ T cells, and neutrophils. Among the key lncRNAs involved, NEAT1, HOTAIR, and TUG1 are crucial in regulating fibroblasts. NEAT1 influences fibroblast activation and proliferation by modulating the production of inflammatory cytokines [173], while HOTAIR affects gene expression related to tissue remodeling and fibroblast activation through its interaction with chromatin-modifying complexes [174]. TUG1 further contributes by regulating genes involved in extracellular matrix production, interacting with transcription factors and chromatin modifiers [175].

In CD8+ T cells, lncRNAs such as GAS5, MALAT1, and ANRIL are essential. GAS5 regulates the proliferation and apoptosis of these cytotoxic T cells, with dysregulated expression exacerbating alveolar destruction in COPD [176]. MALAT1 modulates inflammatory signaling pathways and cytokine production, influencing CD8+ T cell function [177], while ANRIL affects inflammation and cell proliferation through its interaction with chromatin-modifying complexes [178]. Neutrophil activity is regulated by NEAT1, MALAT1, and MEG3. NEAT1 enhances neutrophil recruitment and activation by regulating chemotactic factors like IL-8, whereas MALAT1 modulates neutrophil infiltration and inflammatory cytokine production [179]. MEG3 impacts neutrophil activity by regulating inflammatory cytokines and proteases, affecting transcription factors such as NF-κB [180].

Cigarette smoke and various irritants are primary instigators of COPD, igniting inflammatory cascades within the respiratory tract [181]. Activated macrophages liberate potent neutrophil chemotactic factors like interleukin-8 (IL-8) and leukotriene B4 (LTB4), priming the groundwork for cellular recruitment and activation [182]. Neutrophils, once mobilized, unleash proteases that inflict damage upon the lung parenchyma, fostering emphysematous changes and exacerbating mucus hypersecretion [183]. lncRNAs such as NEAT1 and MALAT1 influence the balance between proteases and inhibitors, including alpha-1-antitrypsin, secretory leukocyte protease inhibitor (SLPI), and tissue inhibitors of metalloproteinases (TIMPs), thereby modulating the dynamic interplay governing tissue homeostasis [184]. Additionally, cytotoxic T cells (CD8+) and fibroblasts orchestrate alveolar wall destruction and fibrotic remodeling under the influence of growth factors released by macrophages and epithelial cells [185].

Inflammation in COPD is a complex phenomenon orchestrated by a myriad of activated inflammatory and structural cells. Lipid mediators such as LTB4, alongside chemokines including monocyte chemotactic protein-1 (MCP-1) and macrophage inflammatory protein-1 alpha (MIP-1α), emerge as pivotal in orchestrating leukocyte recruitment and activation [186]. Interleukin-8 (IL-8), growth-related oncogene alpha (GRO-α), and interferon-gamma-inducible protein-10 (IP-10) further potentiate this cellular influx, attracting cytotoxic T cells and generating reactive oxygen species (ROS) and nitric oxide (NO). The pro-inflammatory cytokine tumor necrosis factor-alpha (TNF-α) assumes a central role in amplifying inflammation, gene expression, and mediating systemic sequelae of COPD [187]. lncRNAs such as HOTAIR and XIST modulate these inflammatory mediators and their downstream effects [188]. Endothelin and transforming growth factor-beta (TGF-β) are pivotal mediators in fibrotic remodeling, epitomizing the intricate interplay between inflammation and tissue remodeling in COPD pathogenesis [189]. The release of proteases, including neutrophil elastase, proteinase C, cathepsins, and MMPs, underscores the proteolytic milieu perpetuating elastolysis and mucus hypersecretion, thus perpetuating the hallmark pathophysiology of COPD [190].

In the complex inflammatory milieu of COPD, lncRNAs intricately modulate the immune response across various contributing cell types. NEAT1, prominently expressed in epithelial cells, neutrophils, and macrophages, regulates IL-8 production, thereby influencing neutrophil recruitment and inflammatory responses [191]. MALAT1 is highly versatile, modulating IL-8, ROS, and NO in epithelial and neutrophil cells, while also affecting the expression of MCP-1, MIP-1α, GRO-α, IP-10, and GM-CSF in endothelial cells, orchestrating monocyte and neutrophil survival and activation. Additionally, MALAT1 impacts the macrophage-driven production of LTB4, contributing to the overall inflammatory cascade [192]. HOTAIR regulates TGF-β and TNF-α production in fibroblasts and macrophages, playing a pivotal role in fibrosis and systemic inflammation [193]. MEG3 influences TGF-β production in fibroblasts and macrophages and modulates MCP-1, MIP-1α, and IL-8 expression, affecting monocyte recruitment and fibrotic responses [194]. GAS5, by modulating ROS, NO, and GM-CSF in epithelial, neutrophil, and macrophage cells, impacts inflammatory responses and neutrophil survival. Furthermore, TUG1’s regulation of neutrophil elastase, proteinase, cathepsins, and MMPs underscores its significant role in tissue remodeling and elastolysis, highlighting its importance in neutrophil-mediated proteolytic activity [195].

Collectively, these lncRNAs orchestrate a complex network of cellular interactions that drive the inflammatory and immune responses in COPD. Their regulation of fibroblasts, CD8+ T cells, neutrophils, and other immune cells underscores their potential as therapeutic targets for mitigating disease progression, emphasizing the critical roles of NEAT1, MALAT1, HOTAIR, MEG3, GAS5, and TUG1 in the pathogenesis and progression of COPD (Figure 4B).

### 4.3. Modulation of Cellular Processes by LncRNA in COPD

LncRNAs play pivotal roles in modulating cellular processes implicated in COPD, contributing to disease pathogenesis and progression. Through diverse mechanisms, including regulation of gene expression, modulation of signaling pathways, and interaction with cellular components, lncRNAs influence key cellular processes in COPD [197].

Epithelial-to-Mesenchymal Transition (EMT): EMT is a cellular process characterized by the transition of epithelial cells into mesenchymal cells, leading to increased cell motility, invasiveness, and fibrosis [198]. Dysregulated expression of lncRNAs such as HOTAIR and MALAT1 has been implicated in promoting EMT in COPD. These lncRNAs regulate the expression of genes involved in EMT-related pathways, including TGF-β signaling and matrix metalloproteinases, facilitating airway remodeling and fibrosis in COPD patients [199].

Cell Proliferation and Apoptosis: Dysregulated cell proliferation and apoptosis contribute to tissue remodeling, airway obstruction, and emphysema in COPD [200]. LncRNAs such as GAS5 and TUG1 modulate cell cycle progression, apoptosis, and senescence in COPD by regulating the expression of genes involved in cell growth and survival pathways. Dysregulated expression of these lncRNAs alters the balance between cell proliferation and apoptosis, contributing to disease progression and exacerbation in COPD patients [201].

Inflammatory Signaling Pathways: Chronic inflammation is a hallmark feature of COPD, characterized by increased production of pro-inflammatory cytokines and chemokines in the lung microenvironment [202]. LncRNAs such as NEAT1 and XIST regulate inflammatory signaling pathways in COPD by modulating the expression of genes involved in cytokine production, immune cell infiltration, and tissue remodeling. Dysregulated expression of these lncRNAs contributes to sustained inflammation and tissue damage in COPD patients, exacerbating disease severity and progression [203].

Oxidative Stress Response: Oxidative stress plays a central role in COPD pathogenesis, contributing to airway inflammation, oxidative damage, and impaired lung function [204]. LncRNAs such as H19 and MEG3 regulate oxidative stress response pathways in COPD by modulating the expression of antioxidant enzymes, stress-responsive genes, and redox signaling molecules. Dysregulated expression of these lncRNAs disrupts cellular homeostasis and exacerbates oxidative damage in COPD patients, contributing to disease progression and exacerbation [205].

Fibrotic Processes: Pulmonary fibrosis is a common complication of COPD, characterized by excessive deposition of extracellular matrix proteins and tissue remodeling in the lung parenchyma [206]. LncRNAs such as FENDRR and HOTAIR regulate fibrotic processes in COPD by modulating the expression of genes involved in fibroblast activation, collagen synthesis, and tissue remodeling. Dysregulated expression of these lncRNAs promotes aberrant fibroblast proliferation and extracellular matrix deposition, leading to progressive fibrosis and loss of lung function in COPD patients [207]. Understanding the molecular mechanisms by which lncRNAs modulate cellular processes in COPD offers insights into disease pathogenesis and potential therapeutic targets for COPD management. Targeting dysregulated lncRNAs may offer novel strategies for inhibiting airway remodeling, inflammation, oxidative stress, and fibrosis in COPD patients, ultimately improving disease outcomes and quality of life [208].

## 5. Modulatory Mechanisms of COPD-Associated LncRNA in Lung Cancer Progression

Lung cancer remains a significant global health challenge, with high mortality rates and limited treatment options. LncRNAs have emerged as critical regulators of lung cancer progression, offering potential insights into novel therapeutic strategies and prognostic markers [209]. This review explores the intricate modulatory mechanisms of lncRNAs in lung cancer progression, focusing on the interaction between COPD-associated lncRNAs and lung cancer pathways, the regulatory functions of lncRNA in lung cancer development from COPD, and the implications of lncRNA dysregulation in lung cancer metastasis [210].

### 5.1. Interaction Between COPD-Associated LncRNA and Lung Cancer Pathways

COPD and lung cancer are two major respiratory diseases that share common risk factors, including cigarette smoking and exposure to environmental pollutants [211]. Emerging evidence suggests that dysregulated lncRNAs play pivotal roles in both COPD pathogenesis and lung cancer progression [212]. This review delves into the intricate relationship between COPD-implicated lncRNAs and lung cancer progression, highlighting the molecular mechanisms underlying their interplay and potential implications for disease management.

#### 5.1.1. Shared Molecular Pathways

COPD-implicated lncRNAs and lung cancer progression often converge on shared molecular pathways involved in inflammation, oxidative stress, and cellular proliferation [213]. For instance, lncRNA H19, initially identified as a regulator of embryonic development and imprinting, exhibits dysregulated expression in COPD and lung cancer tissues [214]. H19 promotes lung cancer progression by modulating pathways associated with cell proliferation, invasion, and metastasis. Its overexpression correlates with advanced tumor stage and poor prognosis in lung cancer patients, highlighting its potential as a prognostic biomarker and therapeutic target [215].

Similarly, lncRNA MALAT1 has been implicated in COPD pathogenesis and lung cancer metastasis. MALAT1 expression is upregulated in COPD and lung cancer tissues, where it promotes tumor invasion and metastasis by regulating alternative splicing events and gene expression programs associated with metastatic progression [216]. Its aberrant expression correlates with disease aggressiveness and poor clinical outcomes in lung cancer patients, underscoring its potential as a therapeutic target for metastatic disease [217].

#### 5.1.2. Regulatory Roles of COPD-Associated LncRNA in Lung Cancer

COPD-implicated lncRNAs exert diverse regulatory functions in lung cancer progression, including modulation of gene expression, epigenetic regulation, and interaction with signaling pathways implicated in tumor development and metastasis [218]. For instance, ANRIL (antisense non-coding RNA in the INK4 locus) is upregulated in COPD and lung cancer tissues, where it promotes tumor growth and metastasis by interacting with chromatin remodeling complexes and transcription factors involved in oncogenic signaling pathways [219].

Moreover, lncRNA TUG1 exhibits dysregulated expression in COPD and lung cancer tissues, contributing to disease aggressiveness and treatment resistance [220]. TUG1 promotes lung cancer progression by modulating epithelial-to-mesenchymal transition (EMT) and metastasis through its interaction with miRNAs and protein-coding genes involved in tumor initiation and progression. Its aberrant expression serves as a prognostic marker for poor survival outcomes in lung cancer patients with a history of COPD, highlighting its potential as a therapeutic target for preventing disease progression [221].

### 5.2. Dysregulated Expression of COPD-Associated LncRNAs in Lung Cancer

COPD is a prevalent respiratory condition characterized by airflow limitation and chronic inflammation, primarily caused by exposure to tobacco smoke and environmental pollutants. It significantly increases the risk of developing lung cancer, the leading cause of cancer-related mortality worldwide. Recent studies have highlighted the dysregulated expression of lncRNAs in both COPD and lung cancer, suggesting their potential roles as biomarkers and therapeutic targets in disease progression [222]. This review aims to explore the dysregulated expression of COPD-associated lncRNAs in lung cancer, elucidating their molecular mechanisms and clinical implications.

ANRIL: ANRIL, also known as CDKN2B-AS1, is a well-characterized lncRNA implicated in COPD and lung cancer pathogenesis [223]. Its dysregulated expression contributes to aberrant cell cycle progression, apoptosis, and cellular senescence, promoting tumor growth and metastasis. In COPD, ANRIL upregulation correlates with disease severity and airflow limitation, suggesting its potential as a biomarker for COPD progression. Similarly, ANRIL overexpression in lung cancer tissues is associated with advanced tumor stage, lymph node metastasis, and poor prognosis in patients [224]. Mechanistically, ANRIL regulates the expression of tumor suppressor genes, oncogenes, and cell cycle regulators, orchestrating a complex network of molecular interactions that drive oncogenic processes in lung cancer cells [225].

H19: H19 is a maternally expressed lncRNA involved in embryonic development and genomic imprinting, whose dysregulated expression has been implicated in COPD and lung cancer progression. In COPD patients, H19 upregulation correlates with airway inflammation, oxidative stress, and lung function decline, suggesting its potential as a diagnostic and prognostic biomarker [226]. In lung cancer, H19 promotes tumor growth, angiogenesis, and metastasis through its interactions with miRNAs, transcription factors, and signaling pathways involved in tumor progression. Its aberrant expression in lung cancer tissues is associated with tumor aggressiveness, treatment resistance, and poor clinical outcomes in patients [227].

MALAT1: MALAT1 is a highly conserved lncRNA implicated in COPD pathogenesis and lung cancer metastasis. Its dysregulated expression contributes to disease progression by modulating alternative splicing events, gene expression programs, and signaling pathways associated with tumor invasion and metastasis [228]. In COPD, MALAT1 upregulation correlates with airway remodeling, mucus hypersecretion, and disease severity, suggesting its potential as a therapeutic target for COPD management. In lung cancer, MALAT1 promotes metastatic spread, EMT, and resistance to chemotherapy and targeted therapies, highlighting its prognostic value and therapeutic potential in advanced-stage disease [229].

TUG1: TUG1 is a conserved lncRNA implicated in COPD pathogenesis and lung cancer progression, whose dysregulated expression correlates with disease severity and poor clinical outcomes. In COPD patients, TUG1 upregulation is associated with airway inflammation, oxidative stress, and lung function decline, indicating its potential as a biomarker for disease progression and exacerbation risk [230]. In lung cancer, TUG1 promotes tumor growth, metastasis, and resistance to therapy by modulating the expression of oncogenes, tumor suppressors, and signaling pathways involved in cell proliferation and survival. Its aberrant expression serves as a prognostic marker for poor survival outcomes in lung cancer patients, highlighting its therapeutic potential as a target for personalized treatment approaches [231].

HOTAIR: HOTAIR is a well-characterized lncRNA implicated in COPD and lung cancer pathogenesis, whose dysregulated expression correlates with disease severity and progression. In COPD patients, HOTAIR upregulation is associated with airway inflammation, fibrosis, and lung function decline, suggesting its potential as a biomarker for disease exacerbation and progression risk. In lung cancer, HOTAIR promotes tumor invasion, metastasis, and angiogenesis by modulating chromatin remodeling complexes, transcription factors, and signaling pathways involved in tumor progression [232]. Its aberrant expression is associated with advanced tumor stage, lymph node metastasis, and poor prognosis in patients, highlighting its prognostic value and therapeutic potential in advanced-stage disease [233].

UCA1: UCA1 is an oncogenic lncRNA implicated in COPD pathogenesis and lung cancer progression, whose dysregulated expression contributes to disease aggressiveness and poor clinical outcomes [234]. In COPD patients, UCA1 upregulation correlates with airway inflammation, mucus hypersecretion, and disease severity, indicating its potential as a biomarker for disease progression and exacerbation risk. In lung cancer, UCA1 promotes tumor growth, metastasis, and resistance to therapy by modulating cell proliferation, apoptosis, and DNA repair mechanisms. Its aberrant expression serves as a prognostic marker for poor survival outcomes in lung cancer patients, highlighting its potential as a therapeutic target for personalized treatment approaches [235].

FENDRR (Fetal-lethal Non-coding Developmental RNA): FENDRR is a developmentally regulated lncRNA implicated in COPD and lung cancer pathogenesis, whose dysregulated expression correlates with disease severity and progression. In COPD patients, FENDRR upregulation is associated with airway inflammation, oxidative stress, and lung function decline, suggesting its potential as a biomarker for disease exacerbation and progression risk [236]. In lung cancer, FENDRR functions as a tumor suppressor by inhibiting cell proliferation, invasion, and metastasis through its interactions with miRNAs and transcription factors involved in tumor progression. Its downregulation is associated with advanced tumor stage, lymph node metastasis, and poor prognosis in patients, highlighting its potential as a prognostic biomarker and therapeutic target in lung cancer [237].

AK098656: AK098656 is a less studied lncRNA but has been implicated in both COPD and lung cancer. Its dysregulated expression suggests potential roles in disease pathogenesis and progression, although further research is needed to elucidate its molecular mechanisms and clinical implications in COPD and lung cancer [238].

## 6. LncRNA in Body Fluids for COPD Diagnosis and Therapy

LncRNAs have emerged as promising biomarkers and therapeutic targets for COPD. Detection of lncRNA biomarkers in body fluids such as blood and urine offers non-invasive diagnostic tools for early disease detection and monitoring [239]. Moreover, the therapeutic potential of lncRNAs in COPD management holds promise for personalized treatment approaches and disease intervention strategies.

### 6.1. Detection of LncRNA Biomarkers in Blood Samples

Blood-based biomarkers have emerged as indispensable tools for diagnosing and monitoring COPD owing to their accessibility and minimally invasive nature. LncRNAs detected in peripheral blood samples have demonstrated considerable promise as diagnostic and prognostic biomarkers for COPD [240]. Among these, NEAT1, MALAT1, and HOTAIR have surfaced as notable candidates, showcasing dysregulated expression patterns in COPD patients compared to healthy controls. Specifically, the circulating levels of these lncRNAs exhibit variations correlated with disease severity, exacerbation risk, and response to therapeutic interventions, underscoring their clinical relevance in the context of COPD diagnosis and prognosis [241].

In recent years, the advent of high-throughput sequencing technologies and sophisticated bioinformatics analyses has accelerated the identification of novel lncRNA biomarkers in blood samples of COPD patients [242]. By leveraging multi-omics data integration and machine learning algorithms, researchers have endeavored to develop robust predictive models for assessing COPD risk and monitoring disease progression. These endeavors hold promise in enhancing the precision and efficacy of COPD management strategies. However, it is imperative to conduct comprehensive validation studies in large patient cohorts to ascertain the diagnostic accuracy and clinical utility of blood-based lncRNA biomarkers in COPD management [243].

Furthermore, the landscape of blood-based biomarkers in COPD extends beyond individual lncRNAs to encompass a spectrum of molecular entities, including miRNAs, messenger RNAs, and proteins. For instance, miR-21 and miR-146a have emerged as key regulators implicated in COPD pathogenesis, displaying dysregulated expression patterns reflective of disease severity and exacerbation risk [244]. Likewise, the expression profiles of certain protein biomarkers, such as C-reactive protein (CRP), interleukin-6 (IL-6), and tumor necrosis factor-alpha (TNF-alpha), exhibit dynamic alterations associated with COPD progression and exacerbation events. The integration of diverse molecular biomarkers holds the potential to furnish clinicians with comprehensive insights into COPD pathophysiology and patient-specific disease trajectories [245].

Moreover, the advent of precision medicine approaches underscores the importance of leveraging blood-based biomarkers to tailor therapeutic interventions according to individual patient profiles. By elucidating the intricate molecular signatures underpinning COPD pathogenesis, blood-based biomarkers pave the way for personalized and targeted therapeutic strategies aimed at mitigating disease progression and improving patient outcomes [246]. The exploration of lncRNA biomarkers in blood samples represents a pivotal avenue in the quest for refined diagnostic and prognostic tools in COPD management. Through concerted efforts in biomarker discovery, validation, and clinical translation, researchers aspire to harness the full potential of blood-based biomarkers to usher in a new era of precision medicine tailored to the intricate nuances of COPD pathophysiology.

### 6.2. Urinary LncRNA as a Diagnostic Tool for COPD

Urine-based biomarkers stand at the forefront of non-invasive and readily accessible samples for diagnosing and monitoring COPD, a prevalent respiratory condition posing significant public health challenges. LncRNAs, discerned within urinary exosomes and cell-free fractions, have emerged as promising candidates for enhancing COPD diagnostic precision [247]. Notably, the dysregulated expression profiles of urinary lncRNAs, including UCA1, TUG1, and H19, have exhibited correlations with the severity of COPD manifestations, declines in pulmonary function, and heightened risks of exacerbations among afflicted individuals. Such associations underscore the potential utility of urinary lncRNAs as indispensable diagnostic biomarkers, offering insights into the dynamic interplay between molecular signatures and disease progression [248].

Delving deeper into the molecular landscape, the characterization of urinary lncRNA profiles in COPD unveils intricate mechanistic underpinnings and unveils potential therapeutic targets. Mechanistic inquiries have shed light on the pivotal roles of urinary lncRNAs in orchestrating inflammatory cascades, modulating oxidative stress responses, and orchestrating tissue remodeling processes within the pulmonary microenvironment. For instance, investigations have delineated how dysregulated urinary lncRNAs, such as UCA1, influence the activation of inflammatory signaling pathways, exacerbating pulmonary inflammation and perpetuating tissue damage characteristic of COPD pathogenesis [249]. Moreover, elucidating the contributions of urinary lncRNAs, like TUG1 and H19, to oxidative stress dynamics underscores their potential as modulators of redox homeostasis, thereby influencing disease progression and exacerbation susceptibility [250].

Furthermore, the exploration of urinary lncRNA signatures unveils novel avenues for personalized treatment strategies and targeted interventions in COPD management. By discerning aberrant expression patterns of urinary lncRNAs, clinicians can tailor therapeutic modalities to address individualized disease phenotypes and molecular profiles [251]. For instance, therapeutic targeting of dysregulated urinary lncRNAs holds promise for mitigating inflammatory responses, ameliorating oxidative stress burdens, and attenuating tissue remodeling processes within the pulmonary microenvironment. Integrating insights from mechanistic studies with clinical observations paves the way for innovative therapeutic approaches aimed at disrupting pathological pathways underpinning COPD progression [252].

In essence, urinary lncRNAs represent invaluable diagnostic tools and therapeutic targets in the multifaceted landscape of COPD. Their intricate roles in mediating inflammatory cascades, modulating oxidative stress dynamics, and orchestrating tissue remodeling processes underscore their significance in unraveling the molecular complexities of COPD pathogenesis [253]. Characterizing the molecular signatures of urinary lncRNAs in COPD provides insights into disease pathogenesis and potential therapeutic targets. Mechanistic studies have elucidated the role of urinary lncRNAs in modulating inflammatory responses, oxidative stress, and tissue remodeling processes in the lung microenvironment. Targeting dysregulated urinary lncRNAs may offer novel strategies for personalized treatment approaches and disease intervention in COPD patients [254]. Harnessing the diagnostic and therapeutic potential of urinary lncRNAs not only enhances our understanding of COPD pathophysiology but also offers unprecedented opportunities for personalized disease management and intervention strategies tailored to individual patient needs [255].

### 6.3. Pharmacological Actions of LncRNA Molecules as Potential Therapeutics for COPD

**Figure 5:**
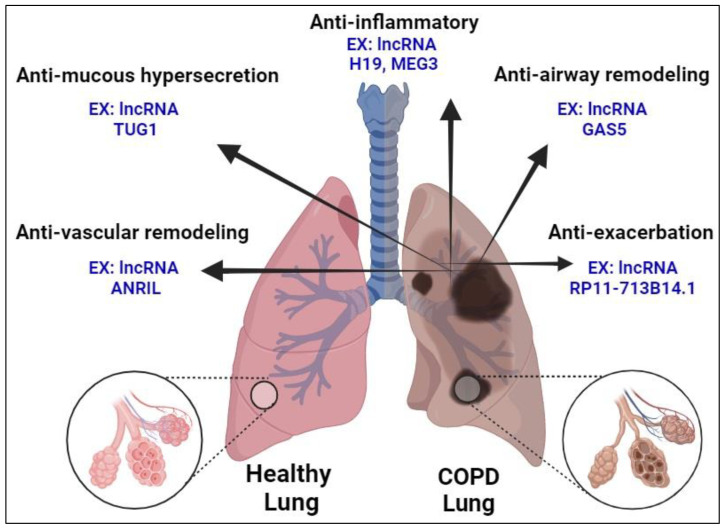
Representative diagram illustrating smoke signals: LncRNAs driving COPD pathophysiology. Tobacco smoke exposure profoundly reshapes the landscape of lncRNAs, instigating a cascade of events leading to exacerbated COPD pathologies. This alteration encompasses mitochondrial dysfunction, chronic inflammation, mucus dysregulation, epigenetic modifications, and cellular senescence. The figure, generated with the BioRender platform, incorporates information derived from reference sources [267].

LncRNAs have emerged as promising targets for pharmacological intervention in COPD, offering potential therapeutic strategies for mitigating disease progression and improving patient outcomes. Several lncRNAs have been identified as key regulators of inflammatory responses, airway remodeling, exacerbation risk, vascular remodeling, and mucous hypersecretion in COPD (Figure 5) [256]. Targeting dysregulated lncRNA molecules holds promise for developing novel therapeutics with anti-inflammatory, anti-airway-remodeling, anti-exacerbation, anti-vascular-remodeling, and anti-mucous-hypersecretion properties [257].

*Anti-inflammatory Actions:* In COPD, chronic inflammation plays a central role in disease pathogenesis, contributing to airway obstruction, tissue damage, and decline in lung function [258]. Dysregulated expression of lncRNAs such as NEAT1, MEG3, H19, MALAT1, and GAS5 has been implicated in modulating inflammatory signaling pathways and cytokine production in COPD patients. Targeting these lncRNAs with pharmacological agents such as antisense oligonucleotides or small interfering RNAs may attenuate inflammatory responses, reduce immune cell infiltration, and mitigate tissue inflammation in COPD [259].

*Anti-Airway-Remodeling Effects:* Airway remodeling is a hallmark feature of COPD, characterized by structural changes in the airway epithelium, smooth muscle hypertrophy, and extracellular matrix deposition [260]. LncRNAs such as GAS5, HOTAIR, TUG1, and FENDRR regulate genes involved in airway remodeling processes, including TGF-β signaling, matrix metalloproteinases, and collagen synthesis. Pharmacological targeting of these lncRNAs may inhibit airway remodeling, restore airway structure and function, and improve lung function in COPD patients [261].

*Anti-Exacerbation Properties:* Exacerbations involve acute worsening COPD symptoms, often triggered by respiratory infections, air pollution, or environmental factors [262]. Dysregulated expression of lncRNAs such as RP11-713B14.1, XIST, UCA1, and H19 has been associated with exacerbation risk and disease progression in COPD patients. Targeting these lncRNAs with pharmacological interventions may reduce exacerbation frequency, attenuate symptom severity, and improve the quality of life of COPD patients [263].

*Anti-Vascular-Remodeling Effects:* Vascular remodeling contributes to pulmonary hypertension, right heart failure, and cardiovascular complications in COPD patients [264]. lncRNAs such as ANRIL, MEG3, HOTAIR, and MALAT1 regulate vascular smooth muscle cell proliferation, endothelial dysfunction, and angiogenesis in COPD. Pharmacological modulation of these lncRNAs may inhibit vascular remodeling, reduce pulmonary vascular resistance, and improve pulmonary hemodynamics in COPD patients [265].

*Anti-Mucous-Hypersecretion Actions:* Mucous hypersecretion is a common feature of COPD, contributing to airway obstruction, impaired mucociliary clearance, and recurrent infections. lncRNAs such as TUG1, GAS5, MALAT1, and NEAT1 regulate mucin gene expression, goblet cell hyperplasia, and mucous production in COPD. Pharmacological targeting of these lncRNAs may reduce mucous hypersecretion, improve airway clearance, and reduce the risk of respiratory infections in COPD patients [266].

### 6.4. Potential Therapeutic Applications of LncRNA in COPD

Therapeutic modulation of lncRNA expression holds promise for COPD management, offering novel approaches for disease intervention and personalized treatment strategies. Targeting dysregulated lncRNAs involved in COPD pathogenesis, such as HOTAIR, MALAT1, and GAS5, may attenuate airway inflammation, oxidative stress, and tissue remodeling in COPD patients [268].

Various strategies have been explored for lncRNA-based therapeutics, including antisense oligonucleotides, small interfering RNAs, and CRISPR-based genome editing. These approaches enable targeted modulation of lncRNA expression and activity, restoring cellular homeostasis and attenuating disease progression in COPD [269]. Moreover, nanoparticle-mediated delivery systems offer efficient and targeted delivery of lncRNA therapeutics to the lung microenvironment, minimizing off-target effects and enhancing therapeutic efficacy [270].

Clinical translation of lncRNA-based therapeutics requires rigorous preclinical validation and safety assessments in animal models and human clinical trials. The optimization of delivery strategies, dosage regimens, and treatment protocols is essential for maximizing therapeutic efficacy and minimizing potential adverse effects. Furthermore, elucidating the molecular mechanisms underlying lncRNA-mediated therapeutic effects in COPD provides insights into disease pathogenesis and potential biomarkers for treatment response monitoring [271].

## 7. Conclusions and Future Directions

The exploration of COPD-associated lncRNAs unveils a sophisticated regulatory network deeply embedded in the pathogenesis and progression of the disease, with significant implications for therapeutic interventions. Key lncRNAs such as NEAT1, TUG1, MALAT1, HOTAIR, and GAS5 demonstrate a crucial role in lung cancer progression and serve as powerful prognostic and diagnostic biomarkers. Dysregulated expression profiles of these lncRNAs are closely linked with COPD severity, providing valuable insights into disease prognosis and treatment response. Longitudinal cohort studies have identified distinct lncRNA signatures that correlate with exacerbation risk, lung function decline, and mortality, thus laying the groundwork for personalized therapeutic regimens. These studies highlight the potential of lncRNAs as reliable markers for predicting disease progression and tailoring individualized treatment strategies. Moreover, the diagnostic and prognostic capabilities of these lncRNAs underscore their potential as therapeutic targets for reducing airway inflammation, oxidative stress, and tissue remodeling in COPD. Translating lncRNA-based interventions into clinical practice demands rigorous validation studies and a deeper mechanistic understanding to optimize treatment strategies and refine biomarker discovery. The intricate interplay between lncRNAs and COPD presents new opportunities for precision medicine, promising improved patient outcomes and enhanced quality of life in COPD management. Through continued research and clinical application, lncRNAs hold the key to transforming COPD treatment paradigms, ensuring more effective and personalized approaches for managing this complex disease.

## Data Availability

This article includes all data presented in the study, along with Supplementary Figure S1 and Supplementary Excel Files S1 and S2 for reference. These Supplementary Materials provide additional detailed insights and data that support and expand upon the findings discussed in the main text.

## Supplementary Materials

The following supporting information can be downloaded at https://www.mdpi.com/article/10.3390/ijms25169001/s1.
