# Supplementary material for: Decoding LncRNA in COPD: Unveiling Prognostic and Diagnostic Power and Their Driving Role in Lung Cancer Progression
Source: Int J Mol Sci. 2024 Aug 19;25(16):9001. doi: 10.3390/ijms25169001 (PMC11354875; doi:10.3390/ijms25169001)
Supplement: Supplementary file 1 [file ijms-25-09001-s001.zip › Figure S1.pdf]

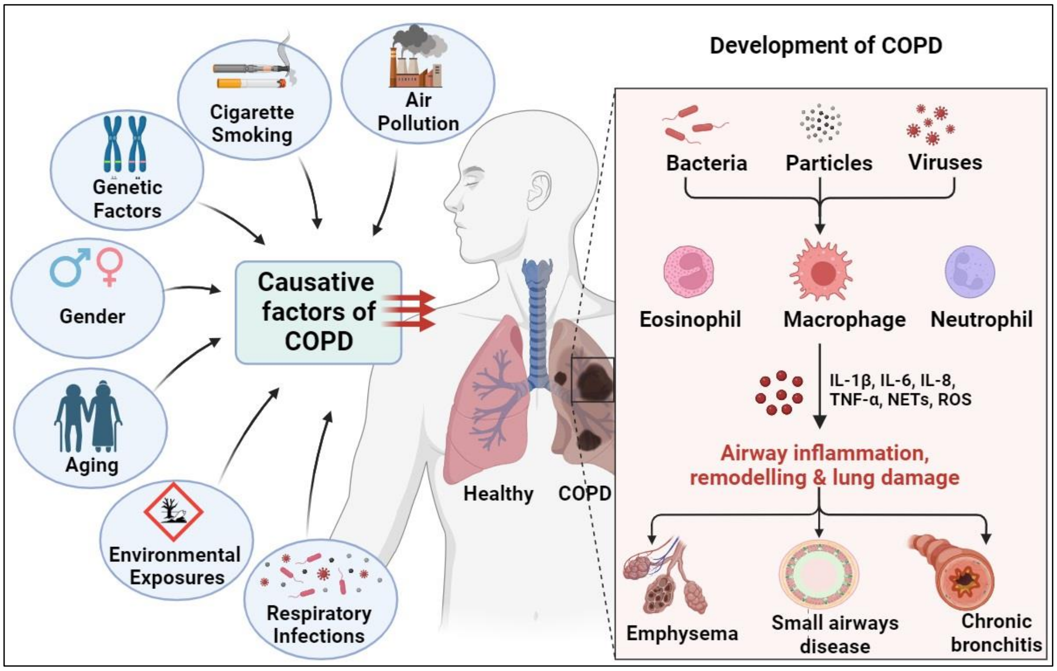


**Figure S1.** The Causative Factors of COPD. Foremost among these is cigarette smoking, a primary contributor to COPD pathology, compounded by environmental exposures and the deleterious effects of air pollution. Genetic predispositions, respiratory infections, and the inexorable process of aging further exacerbate susceptibility. Additionally, socioeconomic disparities and gender differentials intricately intersect, shaping the complex landscape of COPD development. Moreover, the presence of risk factors can activate immune cells, prompting them to initiate airway inflammation, instigate remodeling processes, and inflict lasting lung damage, amplifying the intricate pathogenesis of COPD. The figure, created using BioRender tools, integrates information sourced from the literature review.
